# Radiomics-based discrimination of coronary chronic total occlusion and subtotal occlusion on coronary computed tomography angiography

**DOI:** 10.1186/s12880-024-01248-x

**Published:** 2024-04-09

**Authors:** Jun Li, Lichen Ren, Hehe Guo, Haibo Yang, Jingjing Cui, Yonggao Zhang

**Affiliations:** 1 Department of Radiology, the First Affiliated Hospital of Zhengzhou University, Jianshe East Road, Zhengzhou, Henan 450000 China; 2 Cardiology, The First Affiliated Hospital of Zhengzhou University, Zhengzhou, China; 3 United Imaging Intelligence (Beijing) Co., Ltd, Yongteng North Road, Beijing, 100094 China

**Keywords:** Radiomics, Chronic total occlusion, Subtotal occlusion, Coronary computed tomography angiography

## Abstract

**Objectives:**

Differentiating chronic total occlusion (CTO) from subtotal occlusion (SO) is often difficult to make from coronary computed tomography angiography (CCTA). We developed a CCTA-based radiomics model to differentiate CTO and SO.

**Methods:**

A total of 66 patients with SO underwent CCTA before invasive angiography and were matched to 66 patients with CTO. Comprehensive imaging analysis was conducted for all lesioned vessels, involving the automatic identification of the lumen within the occluded segment and extraction of 1,904 radiomics features. Radiomics models were then constructed to assess the discriminative value of these features in distinguishing CTO from SO. External validation of the model was performed using data from another medical center.

**Results:**

Compared to SO patients, CTO patients had more blunt stumps (internal: 53/66 (80.3%) vs. 39/66 (59.1%); external: 36/50 (72.0%) vs. 20/50 (40.0%), both *p* < 0.01), longer lesion length (internal: median length 15.4 mm[IQR: 10.4-22.3 mm] vs. 8.7 mm[IQR: 4.9-12.6 mm]; external:11.8 mm[IQR: 6.1-23.4 mm] vs. 6.2 mm[IQR: 3.5-9.1 mm]; both *p* < 0.001). Sixteen unique radiomics features were identified after the least absolute shrinkage and selection operator regression. When added to the combined model including imaging features, radiomics features provided increased value for distinguishing CTO from SO (AUC, internal: 0.772 vs. 0.846; *p* = 0.023; external: 0.718 vs. 0.781, *p* = 0.146).

**Conclusions:**

The occluded segment vessels of CTO and SO have different radiomics signatures. The combined application of radiomics features and imaging features based on CCTA extraction can enhance diagnostic confidence.

**Supplementary Information:**

The online version contains supplementary material available at 10.1186/s12880-024-01248-x.

## Introduction

Chronic total occlusion (CTO) is the presence of a complete occlusion in the artery for a minimum of 3 months without any antegrade flow filling indicated by coronary angiography, which is prevalent among patients with ischemic heart disease [1, 2]. Subtotal occlusion (SO) is severe coronary artery stenosis with the positive flow in the distal segment without complete occlusion [3]. Distinguishing between CTO and SO before percutaneous coronary intervention (PCI) is clinically relevant because CTO lesions are more difficult to procedure and have a higher rate of late restenosis compared to non-CTO lesions. However, both conditions can present a complete disruption of luminal blood flow on CCTA imaging, making discrimination between the two conditions difficult. Li et al. [4] and Choi et al. [3] proposed differentiating CTO and SO based on reverse attenuation gradient (RAG) sign, lesion length, blunt stump and collateral vessels. However, this method largely depends on the type of CCTA scanner used and the radiologist’s experience [5–7]. Thus, searching for simple and easily available indicators for differentiating CTO from SO is required.

Radiomics is a relatively new approach in medicine that uses artificial intelligence-driven analytics to extract and convert digital images into mineable and high-dimensional data for extracting quantitative image features in a high-throughput manner, followed by data analysis to support clinical decision-making [8]. Radiomics in cardiovascular diseases has recently received much attention, e.g., for identifying features of high-risk plaques, as well as predicting myocardial ischemia and other coronary artery disease [9–12]. A new study shows that a radiomics model can predict the success of percutaneous coronary intervention [13]. However, there have been no studies on the preoperative application of radiomics to differentiate CTO from non-CTO.

The aim of this study was to develop a diagnostic model to differentiate CTO and SO using non-invasive CCTA imaging-based radiomics.

## Methods

### Study population

The study was approved by local ethics committee (Ethics Number: 2021-KY-0043-002). The ethics committee waived the need for informed consent.

For the internal sets, we retrospectively included 618 patients with CTO or SO who underwent both CCTA and invasive coronary angiography (ICA) from January 2020 to December 2021. The exclusion criteria were as follows: (a) patients who underwent bypass surgery or percutaneous coronary intervention (PCI) for occluded arteries; (b) more than 2 weeks between CCTA examination and ICA examination; (c) the presence of multiple occlusive lesions; (d) too much calcification to accurately assess the lumen; (e) poor image quality. A case-control study was conducted using 1:1 propensity score matching (PSM) to reduce case-control selection bias. The 1:1 PSM used a nearest neighbor matching algorithm for age, gender, BMI, risk factors (hypertension, diabetes, smoking) to reduce the bias in selecting the case controls [14–17].

**Fig. 1:**
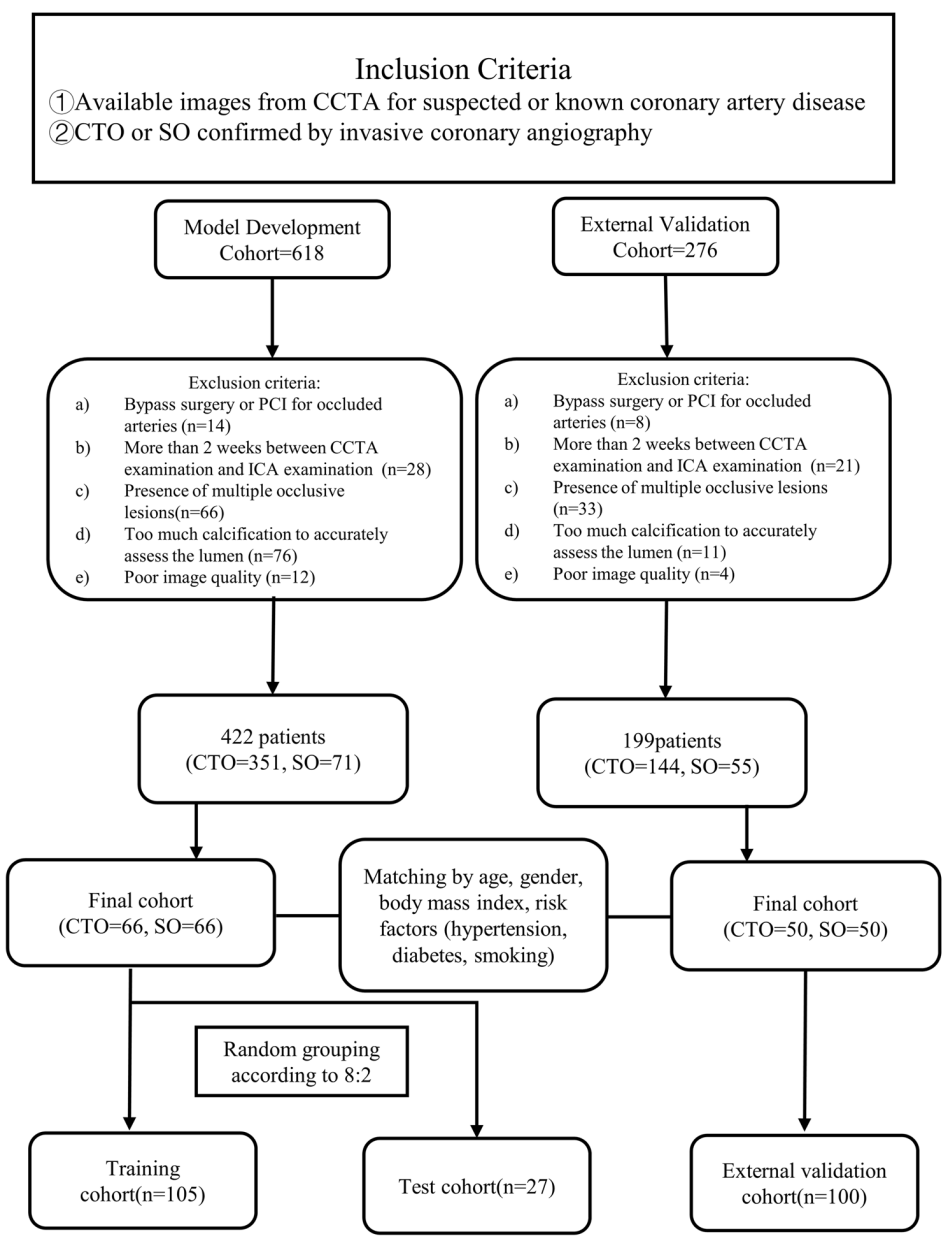
Flow chart of the study design

For the external validation set, we retrospectively recruited 50 patients with CTO and SO each who underwent both ICA and CCTA between January 2017 and October 2022 from Shanghai General Hospital of Shanghai Jiao Tong University with the same eligibility criteria described previously (Fig. 1).

### ICA and percutaneous coronary intervention procedures

ICA was performed with a radial or femoral percutaneous approach, and at least two orthogonal projections were obtained for each major coronary artery. A completely disrupted lumen with no collateral flow via the arterial lesion [Thrombolysis in Myocardial Infarction (TIMI) flow grade 0] was defined as CTO [18]. SO defined as high degree of stenosis with detectable antegrade flow [4]. ICA findings were used as a reference standard to validate CCTA results. Procedure success was defined as the successful opening of total occlusion and restoration of flow (25% residual stenosis and TIMI grade 3 flow) after stent implantation [19]. PCI failure was defined as: (a) failed to cross guidewire, balloon, or stent through occlusion; (b) occurrence of severe complications (including perforation, pericardial tamponade, or hemodynamic instability) that needed emergent intervention during the procedure; (c) PCI operator believed that prolongation of the procedure would not benefit the patient [20].

### CCTA acquisition

For the internal and external test set, CCTA was performed with three CT scanners [a second-generation dual-source CT scanner (SOMATOM Definition Flash; Siemens Healthineers), a third-generation dual-source CT scanner (SOMATOM Force; Siemens Healthineers) and a 256-row wide-detector CT scanner (Revolution HD; GE Healthcare)]. Retrospective ECG-gated CTA was performed in a second-generation dual-source CT, and prospective ECG-triggered acquisition was performed in a third-generation dual-source CT, both of them applying automated tube voltage and current modulation. For wide-detector CT scanner, prospective ECG-triggered acquisition was performed within one heartbeat, and application of automated tube voltage and current modulation. Details of CCTA protocols are presented in Supplemental material.

### CCTA image analysis

CCTA images were imported in the DICOM format into the GE advantage workstation volume share 7 for further analysis. Several CT features which were reported to be indicators for differentiating CTO and SO in a previous study were selected for model development [3]. The lesion length was measured on the planar curve reformation (CPR) images for identifying segments devoid of luminal enhancement. The morphology of the entry point on the angiogram was classified based on the shape of the occluded segment and identified as “tapered” if the occluded segment ended in a funnel shape, otherwise as “obtuse” [21]. We visualized collateral vessels on the CPR images and best-projected three-dimensional maximum intensity projection (MIP) images. The CPR images of intact vascular connections between donor and recipient coronary arteries were used for identifying collateral vessels [3]. Transluminal attenuation gradient (TAG)was determined by the change in Hounsfield units per 10 mm of coronary artery length. TAG (HU/10 mm) was defined by the linear regression coefficient between intra-luminal radiological attenuation and the vessel length from distal to the occlusion [3]. Bending > 45° refers to the bending of the occluded segment at an angle greater than 45° [22]. Proximal and distal side branches were defined as any visible side branch within 3 mm proximal and distal to the occlusion [23]. The ratio of the diameter of the occluded vessel to the adjacent normal vessel > 1 represents positive remodeling [24]. Two radiologists (10 and 5 years of cardiovascular imaging experience, respectively), who were blinded to the ICA findings, independently assessed all CT features. And disagreement was resolved by consensus. Inter-observer agreement for analysis of imaging features was assessed using intraclass correlation co-efficient. Quantitative plaque measurements of the occluded segment vessels were performed on uAI Discover-Coronary (United Imaging Intelligence, Co., Ltd.), and plaque volume and plaque component load were automatically analyzed according to specific thresholds, including calcified plaques (> 350 HU), non-calcified plaques (31 to 350 HU), and low-attenuation plaques (-30 to 30HU).

### Image segmentation and radiomics feature extraction

**Fig. 2:**
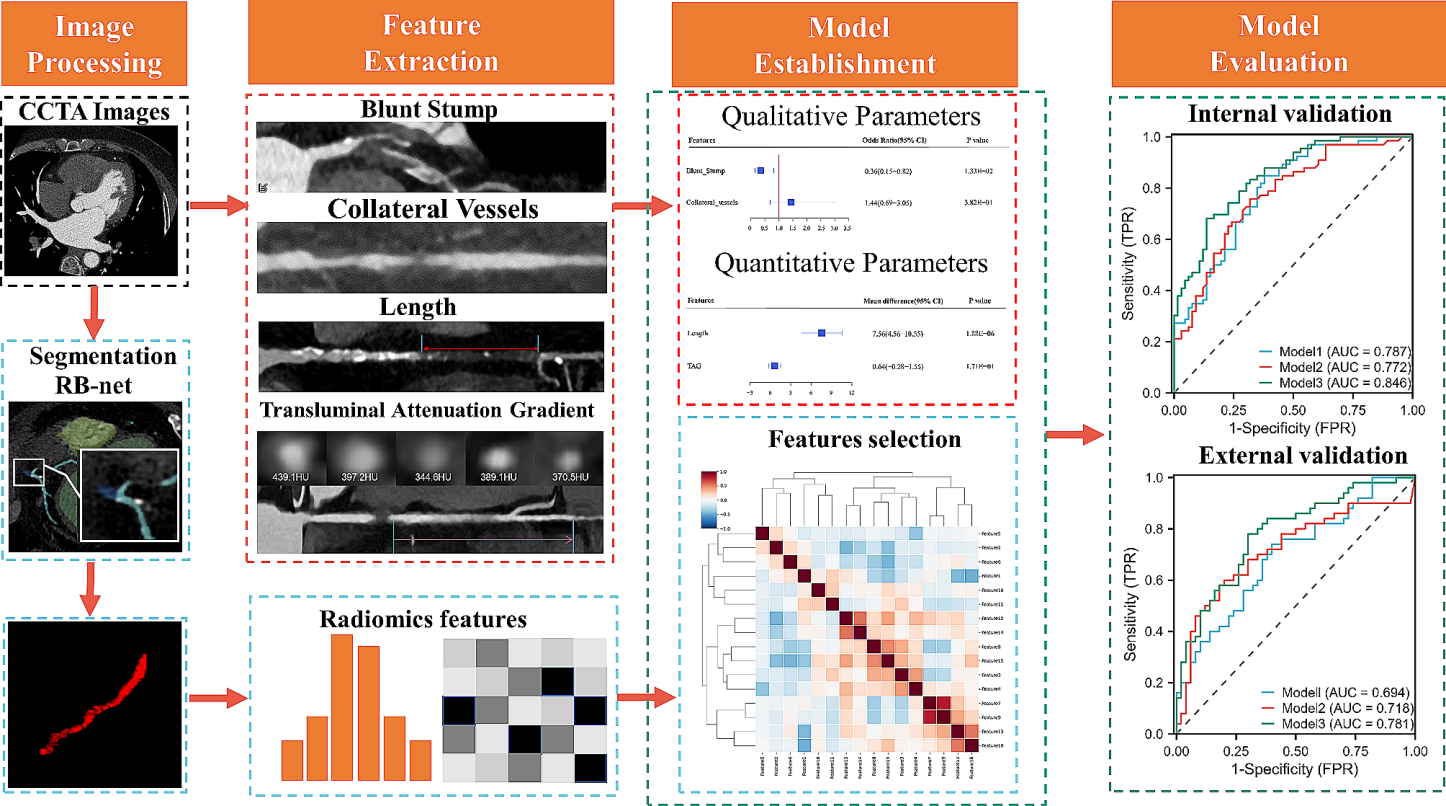
Flow chart showing the process for constructing the prediction models. The red box in the first row represents the imaging model; the blue box in the second row represents the radiomics model; the green box in the last column shows the comparison between the combined model and the other two models

The construction of the automatic segmentation framework and the extraction of radiomics features were performed on Research PortalV1.1 (United Imaging Intelligence, Co., Ltd.). It was integrated with PyRadiomics (https://pyradiomics.readthedocs.io/en/latest). The exact definition of the coronary artery tree was set as a basis for segmenting the occluded segment vessels. For the segmentation of CCTA images of coronary vessel trees, the initial segmentation was performed using the “RB-Net” network. Subsequently, to improve the completeness of the vessel segmentation, the vessel tracking technique was used to connect the broken vessels segmented in the previous step. Additionally, for finer segmentation of the coronary tree, key topological information of the coronary vascular tree was constructed by combining a convolutional graph network with a point cloud network technique [25]. Finally, a bidirectional recurrent convolutional neural network was used to detect the lesion areas of the patients. The region-of-interest (ROI) of the occluded segment was identified and examined by two radiologists. Image preprocessing like wavelet and Laplacian Sharpening filter were performed on all ROIs. Features are divided into 7 groups. Shape features are extracted based on ROI in the original image. Texture features, grayscale statistical features, etc. are extracted from the original image and the filtered image. Finally, 1904 radiomics features were extracted. Detailed categories of features are provided in Supplemental material. The workflow for lumen segmentation and radiomics analysis is shown in Fig. 2.

### Model development and validation

**Fig. 3:**
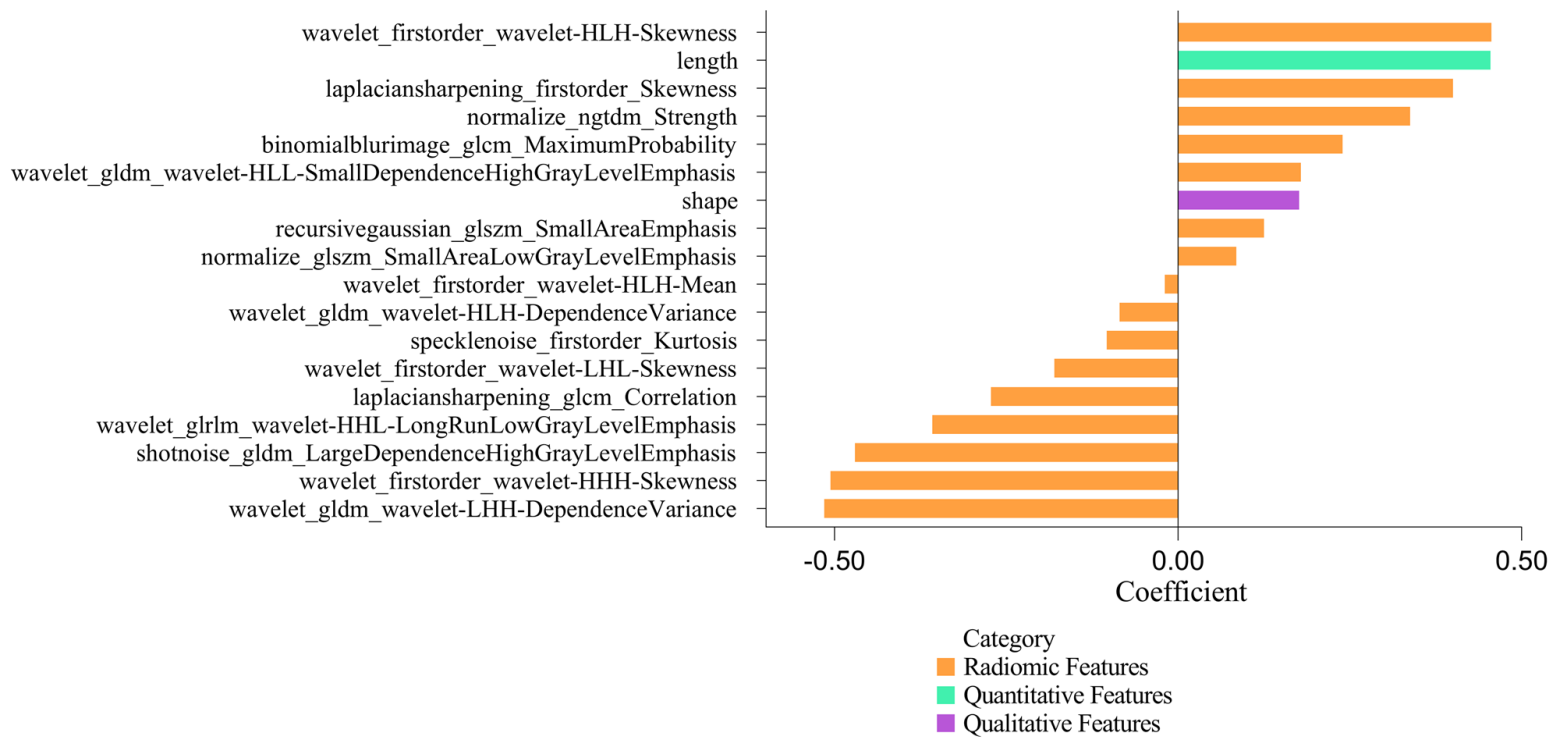
Features coefficient in establishing a combined model

The Z-Score method was used for standardization. First, we performed an ANOVA to screen the radiomics features. Next, LASSO regression analysis was implemented for filtering irrelevant and redundant features. After that, we developed a logistic regression model using the most diagnostic radiomics features (Model 1). Finally, patients were randomly divided in a ratio of 8:2 into the training and test sets. We performed 5-fold cross-validation to demonstrate good agreement among these data (Supplemental Appendix). Next, quantitative and qualitative features obtained from CCTA images were compared to filter out statistical factors and construct imaging models (Model 2). Finally, we merged the radiomics model (Model 1) and the imaging features (Model 2) to construct the combined model (Model 3). The features were ranked according to the coefficient in establishing combined model (Fig. 3). We evaluated the performance of the radiomics-based ML model in an independent study sample as external validation. The CCTA imaging protocol and segmentation of occluded segmental vessels followed the same procedure as described above.

### Statistical analysis

We analyzed the data using “R 4.2.2” software. Fisher’s exact or chi-squared tests were used to compare categorical data. The normality tests were performed on the count data. The independent samples *t*-test was performed on data conforming to a normal distribution, and the Mann-Whitney *U* test was performed on data that did not follow the normal distribution. *P* < 0.05 was considered statistically significant. The receiver operating characteristic (ROC) curve was used to analyze the ability of the imaging, radiomics, and combined models to differentiate between CTO and SO. The DeLong test was used to compare the area under the ROC curve (AUC) values [26]. We plotted the calibration curves to determine the agreement between the observed and predicted results of the three models. Next, we performed the “decision curve analysis (DCA)” to determine the application of the three models in clinical settings by calculating the net benefit at different threshold probabilities [27].

## Results

### Baseline patient characteristics

The basic characteristics of the study population are shown in Table 1, revealing no statistically significant difference in basic characteristics between the two groups. In addition, we compared the differences in the outcomes of PCI between the two groups. Among 66 CTO patients, 17 received no treatment, 5 underwent coronary artery bypass grafting, and 34 PCI was successful (31 with antegrade and 3 retrograde method). Among SO patients, 55 received treatment, 1 coronary artery bypass graft was performed, and 51 PCI was successful (all antegrade). PCI was attempted less frequently (66.7% vs. 81.8%), with a much lower procedural success rate (77.3% vs. 94.4%) in CTO compared with SO (*p* < 0.05). The procedural time was 24.00 min (IQR [7.50–39.50]) in CTO patients and 5.00 min (IQR [2.00-11.50]) in SO patients, with the longer time required for opening in CTO patients (*p* < 0.001). The basic characteristics of the study population in the training set, test set and external validation set are shown in Supplementary Table 1.

**Table 1:** Baseline characteristics of the study population

	SO (*n* = 66)	CTO (*n* = 66)	P Value
Clinical characteristics			
Male	41 (62.1)	50 (75.8)	0.090
Age, y	65 (55–71)	63 (58–73)	0.238
Body mass index, kg/m^2^	24.37 ± 3.45	23.98 ± 3.00	0.483
Hypertension	38 (57.6)	42 (63.6)	0.476
Diabetes	26 (39.4)	22 (33.3)	0.469
Smoking	20 (30.3)	28 (42.4)	0.148
MI	14 (21.2)	20 (30.3)	0.232
Unstable angina	10 (15.2)	16 (24.2)	0.189
Stable angina	14 (21.2)	16 (24.2)	0.678
Silent ischemia	9 (13.6)	10 (15.2)	0.804
PCI			
PCI attempted	54 (81.8)	44 (66.7)	0.047
Successful PCI	51 (94.4)	34 (77.3)	0.013
Procedural time (min)	5.00 (2.00-11.50)	24.0 (7.50–39.50)	< 0.001

Values are mean ± SD, median (25th and 75th percentile) or n (%)

MI = myocardial infarction; PCI = percutaneous coronary intervention

### CCTA image analysis

#### Qualitative parameters

The presence of blunt stump was significantly higher in CTO patients than in the SO group (80.3% vs. 59.1%, *p* = 0.008), but there was no significant difference in the appearance of collateral vessels, proximal branch, distal branch, bending > 45°and positive remodeling between the two groups (40.9% vs. 50.0%, 34.8% vs. 47.0%, 24.2% vs. 28.8%, 21.2% vs. 16.7% and 33.3% vs. 24.2%, all *p* > 0.05) (Table 2). In the external validation group, the proportion of blunt stumps was significantly higher in CTO patients than in SO patients (72.0% vs. 40.0%, *p* < 0.01). There was no significant difference in collateral vessels, proximal branch, distal branch, bending > 45°and positive remodeling between the two groups (52.0% vs. 40.0%, 42.0% vs. 28.0%, 20.0% vs. 16.0%, 32.0% vs. 24.0% and 48.0% vs. 30.0%, all *p* > 0.05).

**Table 2:** CCTA features of the study population

	SO (*n* = 66)	CTO (*n* = 66)	P value
Lesion location			
LAD	28 (42.4)	19 (28.8)	0.102
LCX	8 (12.1)	11 (16.7)	0.457
RCA	30 (45.5)	36 (54.5)	0.296
Lesion length, mm	8.65 (4.88–12.63)	15.35(10.43–22.33)	*<* 0.001
TAG (HU/10 mm)	-1.33 (-2.56 to -0.13)	-0.12 (-2.27 to 0.10)	0.076
Blunt stump	39 (59.1)	53 (80.3)	0.008
Collateral vessel	33 (50.0)	27 (40.9)	0.294
Proximal branch	31 (47.0)	23 (34.8)	0.157
Distal branch	19 (28.8)	16 (24.2)	0.554
Bending > 45°	11 (16.7)	14 (21.2)	0.505
Positive remodeling	16 (24.2)	22 (33.3)	0.249
Total plaque volume (mm^3^)	263.23 (103.00–428.23)	344.15 (185.41–551.46)	0.108
Calcified plaque volume (mm^3^)	54.68 (12.20–196.95)	87.42 (42.30–257.62)	0.188
Non-calcified plaque volume(mm^3^)	169.00 (84.55–266.22)	228.60 (113.93–340.85)	0.063
Low-attenuation plaque volume(mm^3^)	30.80 (7.18–69.94)	49.61 (17.41–88.06)	0.057
Calcified plaque load (%)	32.86 (11.16–52.88)	31.24 (16.58–51.24)	0.931
Non-calcified plaque load (%)	67.14 (47.12–88.84)	68.76 (48.76–82.78)	0.912
Low-attenuation plaque load (%)	10.49 (3.94–22.69)	13.27 (7.43–22.28)	0.251

Values are median (25th and 75th percentile) or n (%)

*Abbreviations* CTO = chronic total occlusion; SO = subtotal occlusion; CCTA = coronary computed tomography angiography

### Quantitative parameters

In both internal and external study samples, lesion lengths were significantly different between the two groups, with CTO patients having longer lesion lengths than SO patients (internal:15.35 mm [IQR 10.43 to 22.33] vs. 8.65 mm [4.88 to 12.63]; external:11.75 mm [6.13 to 23.43] vs. 6.20 mm [3.48 to 9.13], both *p* < 0.001). TAG was not obviously different between both groups (internal: -0.12[-2.27 to 0.10] vs. -1.33[-2.56 to -1.33]; external: -1.14[-2.21 to 0.14] vs. -1.79[-2.97 to -0.82], both *p* > 0.05). There was no significant difference in plaque volume and plaque component load between the two groups (all *p* > 0.05). The CCTA features of the training set, test set and external validation set are shown in Supplementary Table 2. The inter-observer agreement was > 0.75 for all measured parameters (Supplementary Table 3).

### Radiomics analysis

**Fig. 4:**
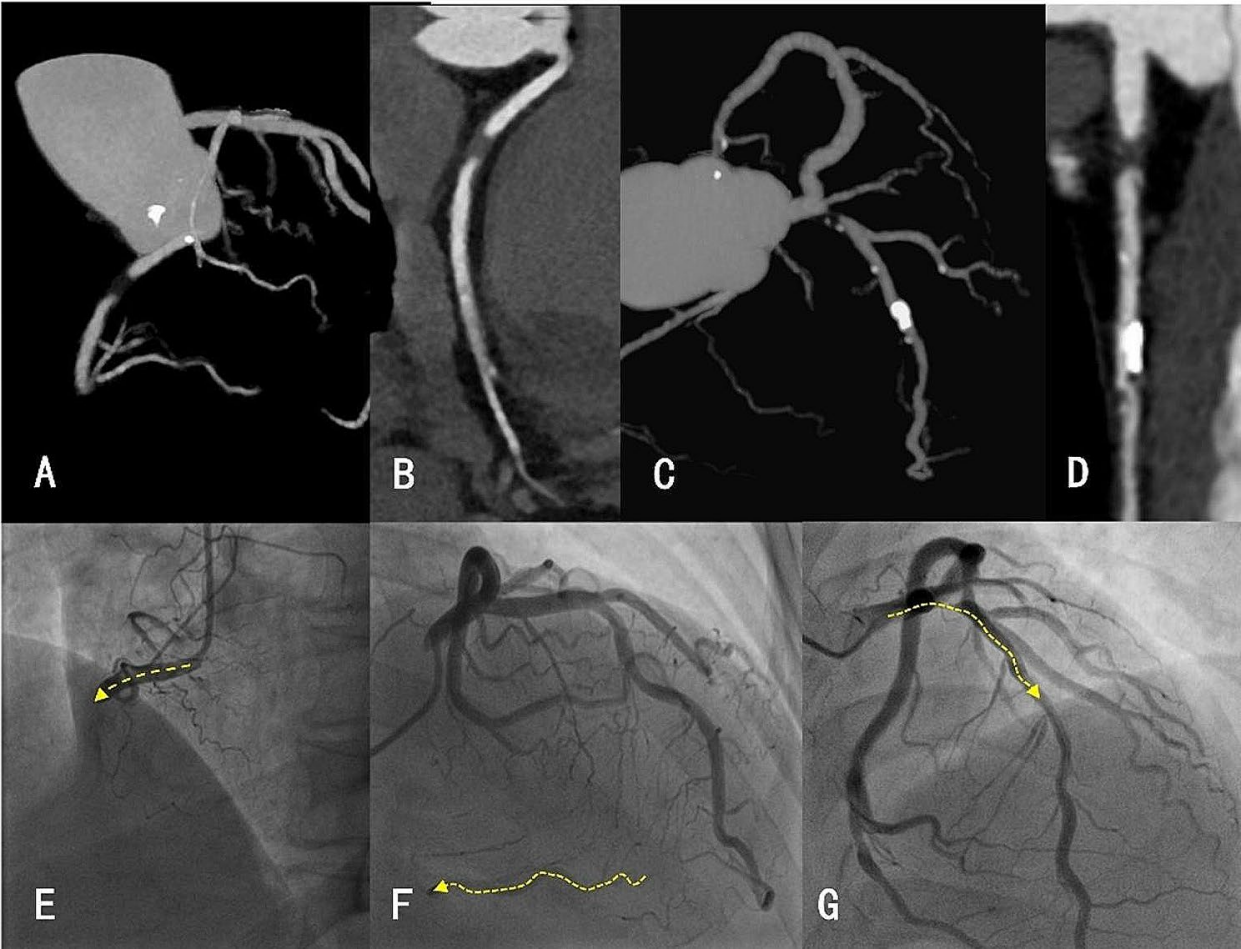
CCTA and DSA diagrams for patients with CTO and SO. (**A, B**) Maximum intensity projection (MIP) and curve planar reformation (CPR) images of patients with CTO;(**C, D**) MIP and CPR images of patients with SO;(**E, F**) Digital subtraction angiography images of a patient with CTO. E shows no positive blood flow far from the occluded segment (yellow arrow); F shows the LAD distal-RCA vessel visualization with LAD-RCA reverse flow (yellow arrow). (**G**) Digital subtraction angiography image of a patient with SO, wherein positive flow was observed far from the stenotic segment (yellow arrow)

We performed an ANOVA to screen the radiomics features, and a total of 55 significantly different features (*p* < 0.05) were screened. Using LASSO regression analysis, 16 radiomics features with significant diagnostic ability were selected. The 16 features include the six first-order features and ten texture features. Distinguishing between CTO and SO was challenging when the length and morphology of the occluded vessels were similar (Fig. 4).

### Performance of model

The AUC values of Model 1 in the training, test, and external validation sets were 0.795 (95% CI: 0.705–0.867), 0.775 (95% CI: 0.574–0.912) and 0.694 (95% CI: 0.594–0.783), respectively. The AUC values of Model 2 in all three sets were 0.771 (95% CI: 0.679–0.848), 0.769 (95% CI: 0.568–0.908), and 0.718 (95% CI: 0.619–0.803). Model 3 demonstrated the best accuracy in predicting CTO or SO, with the AUC values of 0.849 for the training set (95% CI, 0.766–0.911), 0.830 (95% CI, 0.636–0.946) for the test set, and 0.781 (95% CI, 0.687–0.858) for the external validation set (Table 3, Supplementary Fig. S1). The decision curves demonstrate the clinical usefulness of the prediction models by comparing the net benefit at different threshold probabilities in the training and validation sets. (Supplementary Fig. S1). The calibration curves (Supplementary Fig. S2) revealed that all prediction models showed a good fit in all three sets (*P* > 0.05 in the Hosmer-Lemeshow test).

**Table 3:** Comparison of the diagnostic performances among models

cohort		Model 1	Model 2	Model 3
Training set	AUC (95% CI)	0.795(0.705–0.867)	0.771(0.679–0.848)	0.849(0.766–0.911)
	SPE (95% CI)	0.604(0.460–0.733)	0.698(0.555–0.813)	0.849(0.719–0.928)
	SEN (95% CI)	0.865(0.736–0.940)	0.750(0.608–0.855)	0.673(0.528–0.793)
	ACC (95% CI)	0.733(0.730–0.737)	0.724(0.720–0.728)	0.762(0.759–0.765)
	PPV (95% CI)	0.682(0.554–0.788)	0.709(0.569–0.820)	0.814(0.661–0.911)
	NPV (95% CI)	0.821(0.659–0.919).	0.740(0.594–0.849)	0.726(0.596–0.828)
	cut-off	0.435	0.454	0.556
Test set	AUC (95% CI)	0.775(0.574–0.912)	0.769(0.568–0.908)	0.830(0.636–0.946)
	SPE (95% CI)	0.692(0.389–0.896)	0.846(0.537–0.973)	0.846(0.537–0.973)
	SEN (95% CI)	0.929(0.642–0.996)	0.643(0.356–0.860)	0.786(0.488–0.943)
	ACC (95% CI)	0.815(0.804–0.826)	0.741(0.727–0.755)	0.815(0.804–0.826)
	PPV (95% CI)	0.765(0.498–0.922)	0.818(0.478–0.968)	0.846(0.537–0.973)
	NPV (95% CI)	0.900(0.541–0.995)	0.688(0.415–0.879)	0.786(0.488–0.943)
External validation set	AUC (95% CI)	0.694(0.594–0.783)	0.718(0.619–0.803)	0.781(0.687–0.858)
	SPE (95% CI)	0.600(0.452–0.733)	0.800(0.659–0.895)	0.700(0.552–0.817)
	SEN (95% CI)	0.740(0.594–0.850)	0.600(0.452–0.733)	0.780(0.637–0.880)
	ACC (95% CI)	0.670(0.666–0.674)	0.700(0.696–0.704)	0.740(0.736–0.744)
	PPV (95% CI)	0.649(0.511–0.768)	0.750(0.585–0.868)	0.722(0.581–0.831)
	NPV (95% CI)	0.698(0.537–0.823)	0.667(0.532–0.780)	0.761(0.609–0.869)

AUC = area under curve; 95% CI = 95% confidence interval; SPE = specificity; SEN = sensitivity; ACC = accuracy; PPV = positive predictive value; NPV = negative predictive value

## Discussion

The key findings of this study are: (1) the length and blunt stump were the most sensitive metrics imaging metrics to discriminate CTO and SO; (2) the lumen of the occluded segment of CTO showed different radiomics features compared to SO; (3) radiomics can provide support when the length and shape of the occluded segment are essentially identical.

Differential diagnosis of CTO and SO is clinically important. CTO predicts a more difficult procedure, lower success rate, higher complication rate, higher radiation exposure and longer procedure time for PCI than non-CTO [2, 28–30]. Identification of CTO and SO is probably useful in estimating the difficulty of the procedure or deciding on a revascularization strategy. CCTA is a non-invasive method of assessing coronary artery disease and is recommended as a valuable preoperative imaging tool for CTO [31].The ability of CCTA to detect CTO may guide more specialized personnel device selection prior to the procedure. Thus, acquiring CCTA image information allows cardiologists to focus on selecting and performing the required procedures without wasting time on diagnosis [5]. Currently, various CCTA-based clinical evaluation indexes, such as the lesion length, calcification area, presence of blunt stump, and intra-luminal attenuation gradient, are used to differentiate CTO and SO [3, 4]. However, the acceptable threshold values for the lesion length and TAG are still lacking. Because the non-wide-body detector CT scanner uses a prospective scanning method, differences in the contrast concentration occur in different axial images, which might affect the accuracy of the TAG values [32]. Additionally, differences in the clinician approach increase the difficulties in assessing TAG and collateral vessels. This also explains why only lesion length and blunt stump resulted to be independent predictors in our study, from which the CCTA imaging model is constructed.

Novel image and data analytic techniques such as radiomics, machine learning (ML), and deep learning(DL) may decrease inter-reader variations, increase the amount of quantitative information, and improve diagnostic and prognostic accuracy while reducing subjectivity and biases [33]. CCTA provides a platform for linking radiomics to clinical medicine, as it is widely used to diagnose coronary-related diseases because of its low acquisition and post-processing requirements and a large amount of easily available data [34]. Radiomics can provide information that cannot be perceived quantitatively by human eyes, enhancing our understanding of diseases and ultimately aiding clinical decision-making. The advent of radiomics allows inexperienced clinicians to quickly identify differences that are difficult to distinguish visually. This technique has several benefits, like quick and easy to perform, and requires no additional trauma, exposure to radiation, scanning time, or the contrast agent [35]. Therefore, our study assists in the manual differentiation of CTO and SO for diagnosis using radiological features, feature selection and construction of predictive models based on machine learning methods.

Recent reports have shown that deep learning models can significantly reduce the post-processing time for CTO quantification on CCTA images compared to traditional manual reconstruction. The occlusion features based on the deep learning model have excellent correlation and consistency compared to the anatomical assessment of manual reconstruction [25]. Previous radiomics studies on coronary artery disease have focused on the plaque component, peri-coronary adipose tissue or myocardium of the coronary arteries; however, our study first reported using radiomics to a more precise coronary lumen, which may enable the discovery of pathological heterogeneity between CTO and SO. CTO lesions are thrombotic occlusions with fibrous tissues rich in collagen or calcification of the lumen of the occluded segment [36, 37], whilst SO as an incomplete occlusion. Differentiating CTO and SO based on subjective assessment of CCTA images without considering the complex spatial relationships between voxels may result in the loss of important information. 10 of the 16 features extracted in this study were texture features containing voxels, and the highest coefficient value for model importance was also for texture features. It may potentially reflect differences in pathology, which side-steps the ability of radiomics to discriminate CTO from SO. wavelet_gldm_wavelet-LHH-DependenceVariance and shotnoise_gldm_LargeDependenceHighGrayLevelEmphasis are the two texture features with the highest model coefficient values which respond to the dependence of the gray values in the image. Higher values of the above features indicate that the occluded segment of the vessel has a large dependence of higher gray values, which may be related to the fact that the CTO is enriched with more fibrocalcified tissue [29, 38].

In this study, we constructed a combined model based on radiomics data and CCTA imaging features of the lumen of the occluded coronary segment. The performance of the combined model in diagnosing CTO from SO was better compared to the imaging and radiomics models, indicating that the former could help overcome the differences in the diagnostic ability of different scanning modalities and inexperienced doctors, resulting in improved diagnostic accuracy. Therefore, the radiomics features based on extracting CCTA images can accurately and reliably distinguish between CTO and SO before PCI and aid clinical decision-making.

The key limitations of this study should be considered. First, this was a retrospective study with small sample size. Second, the perivascular information of the occluded segment was not incorporated because the outlined ROI was located in the lumen, which could result in the loss of some valuable information. Future studies with large sample sizes and prospective designs are needed to validate the model generalizability.

In conclusion, this study develops a diagnostic model to differentiate CTO and SO using non-invasive CCTA imaging-based radiomics that can provide support when the CCTA metrics are similar. Our study would help interventional cardiologists predict the ease of percutaneous coronary intervention. Future studies should assess the value of the radiomics features for guiding treatment.

### Electronic supplementary material

Below is the link to the electronic supplementary material.

Supplementary Material 1

## Data Availability

The datasets used and/or analyzed during the current study are available from the corresponding author on reasonable request.

## Nonstandard Abbreviations and Acronyms

CCTA: Coronary Computed Tomography Angiography
TAG: Transluminal Attenuation Gradient
CTO: Chronic Total Occlusion
SO: Subtotal Occlusion
ICA: Invasive Coronary Angiography
PCI: Percutaneous Coronary Intervention
LASSO: Least Absolute Shrinkage and Selection Operator
ANOVA: Analysis of Variance
