# Supplementary material for: Radiomics-based discrimination of coronary chronic total occlusion and subtotal occlusion on coronary computed tomography angiography
Source: BMC Med Imaging. 2024 Apr 9;24:84. doi: 10.1186/s12880-024-01248-x (PMC11005149; doi:10.1186/s12880-024-01248-x)
Supplement: Supplementary file 1 — Supplementary Material 1 [file 12880_2024_1248_MOESM1_ESM.docx]

**Supplemental Methods**

**CCTA acquisition**

For the internal and external test set, CCTA was performed with three CT scanners [a second-generation dual-source CT scanner (SOMATOM Definition Flash; Siemens Healthineers), a third-generation dual-source CT scanner (SOMATOM Force; Siemens Healthineers) and a 256-row wide-detector CT scanner (Revolution HD; GE Healthcare)]. Retrospectively ECG-gated CTA was performed in second-generation dual-source CT, with a reconstructed slice thickness of 0.6 mm, reconstructed slice interval of 0.5mm, and application of automated tube voltage and current modulation (CARE Kv, CARE Dose 4D, Siemens Healthineers, Germany). Prospective ECG-triggered acquisition was performed in third-generation dual-source CT, with a reconstructed slice thickness of 0.75 mm, reconstructed slice interval of 0.5mm, and application of automated tube voltage and current modulation (CARE Kv, CARE Dose 4D, Siemens Healthineers, Germany). For both dual-source CT scans, the images were reconstructed using a vascular algorithm (Bv36f) and Iterative Reconstruction (ADMIRE; Siemens) with a strength level of 3. Prospective ECG-triggered acquisition was performed in wide-detector CT scanner within one heartbeat, with a reconstructed slice thickness of 0.625 mm, and application of automated tube voltage and current modulation (KV Assist, Smart mA, GE Healthcare, USA). Images were reconstructed using ASiR-V 50%, which implied 50% filtered back projection (FBP) blending with 50% ASiR in reconstructed images.

All patients took 0.5 mg of nitroglycerin sublingually before the examination. All patients were administered 30-58 mL contrast medium, into the vein of the right elbow at a 3.8-5.8 mL/s flow rate and were flushed using 50 mL saline. The amount of contrast and injection rate were determined based on the individual weight and prescribed contrast concentration in order to maintain consistent iodine delivery rate (IDR) across all patients (Supplementary Table 4).

**Image segmentation and radiomics feature extraction**

Features are divided into 7 groups. Shape features are extracted based on ROI in the original image. Texture features, grayscale statistical features, etc. are extracted from the original image and the filtered image. A total of 1904 features were extracted. The categories of features are as follows:

(1)shape：14；

(2)first-order gray-level statistics：378；

(3)Gray Level Cooccurence Matrix (GLCM)：441；

(4)Gray Level Run Length Matrix (GLRLM)：294；

(5)Gray Level Size Zone Matrix (GLSZM)：336；

(6)Neighbouring Gray Tone Difference Matrix (NGTDM)：336；

(7)Gray Level Dependence Matrix (GLDM)：105

For more detailed information please refer to [https://urp.united-imaging.com/getdcm/DOC/RadiomicsFeature.pdf](https://urp.united-imaging.com/getdcm/DOC/RadiomicsFeature.pdf) and <https://urp.united-imaging.com/getdcm/DOC/RadiomicsFilter.pdf>

**Supplemental Figures and Figure Legends**


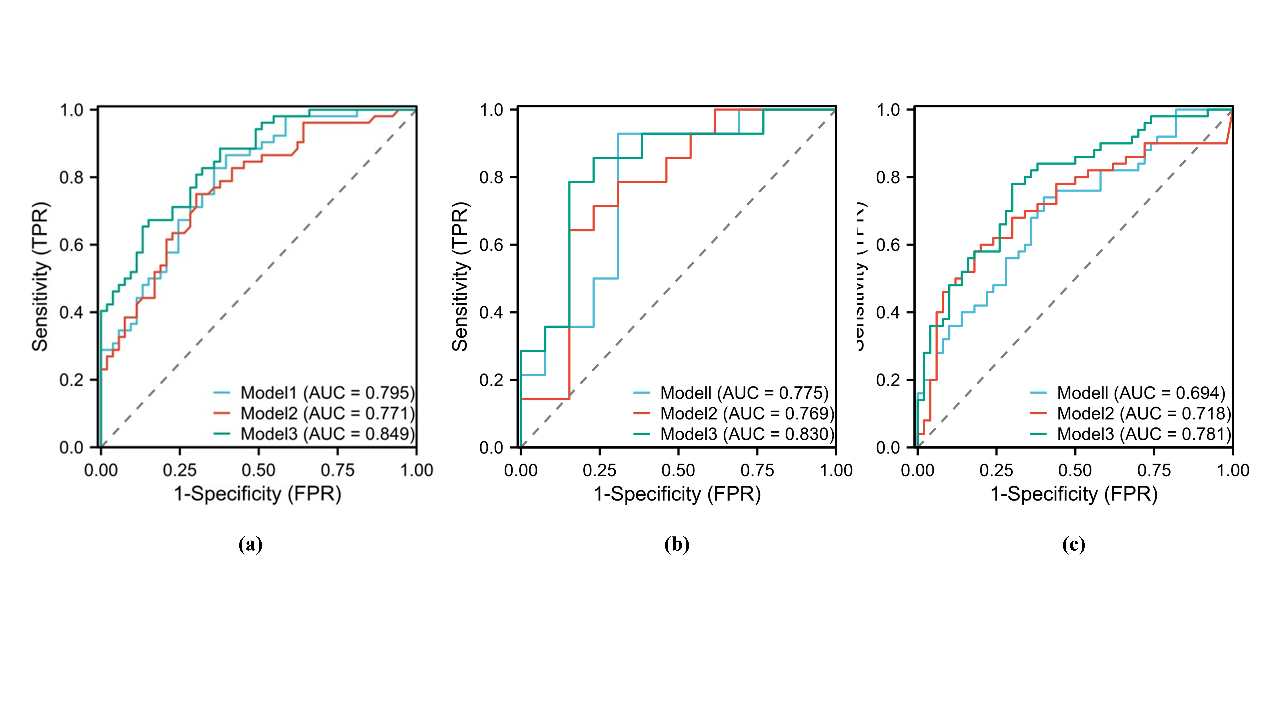


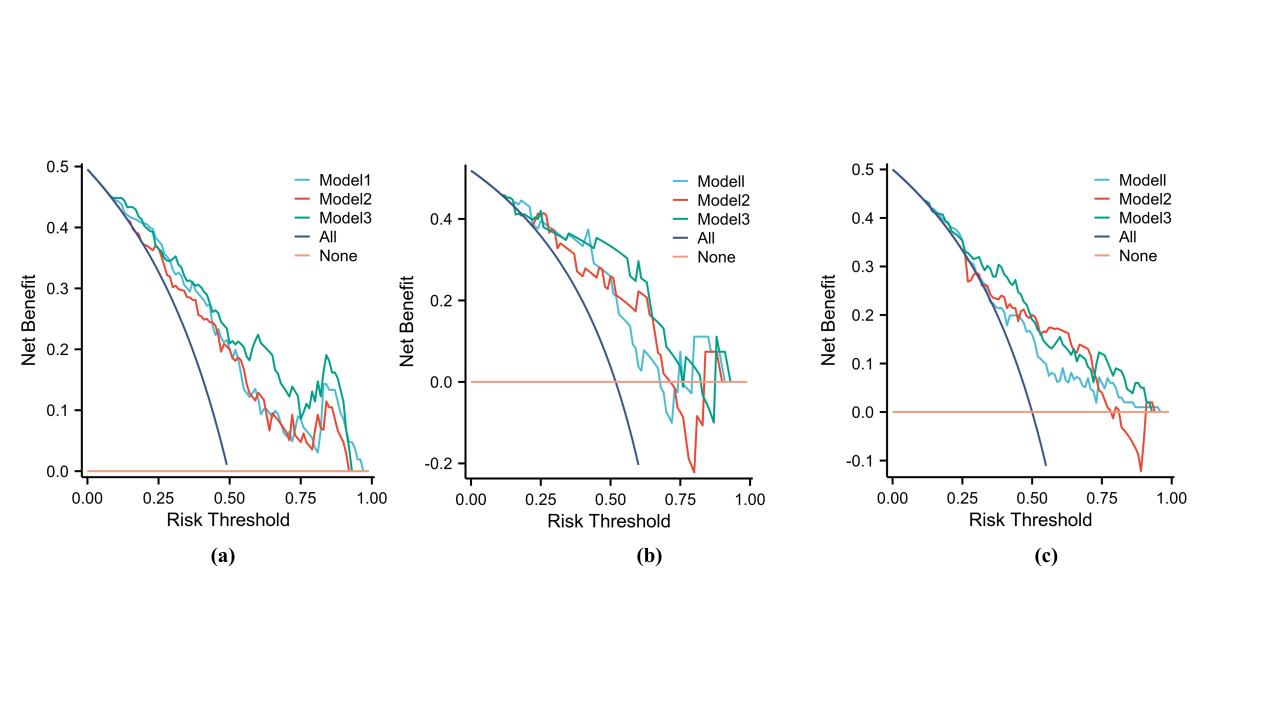


Supplementary Fig. 1 Receiver operating characteristic (ROC) curves and Decision curve analysis Receiver operating characteristic (ROC) curves and Decision curve analysis for the three models in the training (a), test(b) and external validation (c) sets. The combined model had a higher area under the curve (AUC) than the other models. The blue curved line represents the assumption that all patients are CTO, while the orange straight line represents the assumption that no patients are CTO. The combined model had a higher net benefit than the other models.


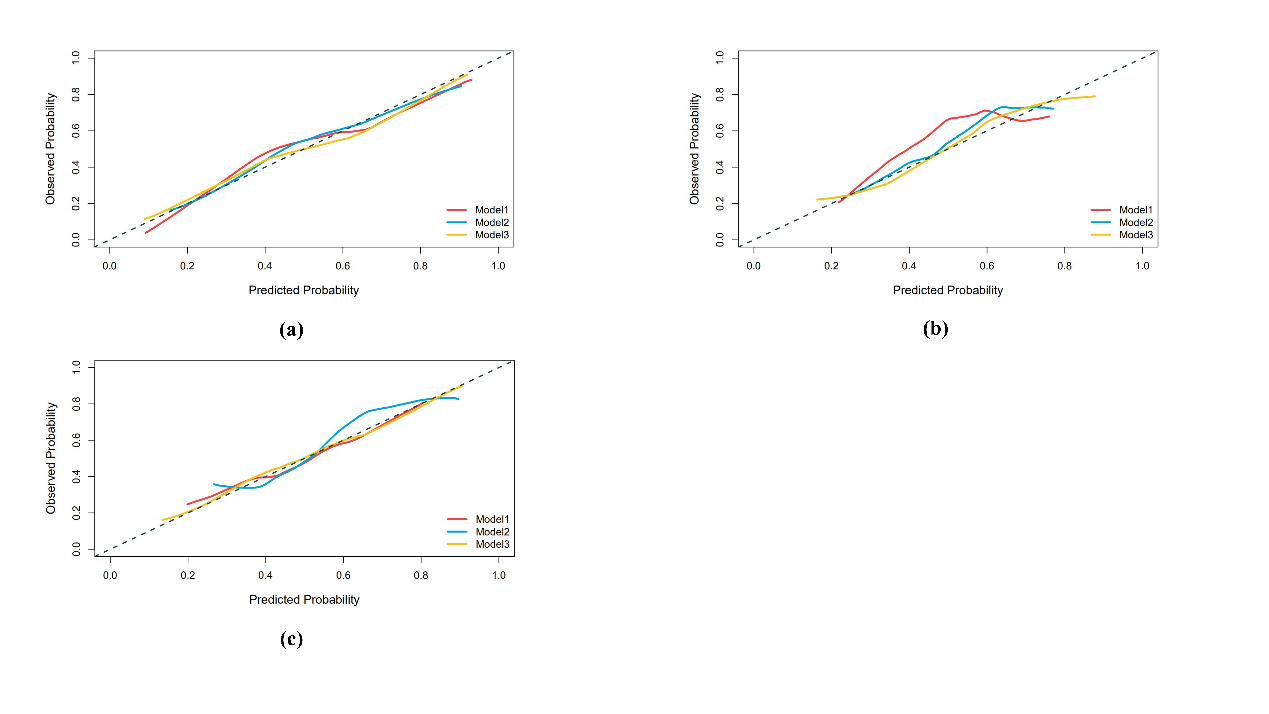


Supplementary Fig. 2 Calibration curve. Calibration curves for the three models in the training (a), test (b) and external validation (c). The calibration curves represented the fitness of predicted probability of the three models to the real outcomes. The combined model has the highest fitness.

**Supplemental Appendix: Five-fold cross-validation ROC, calibration curve, and DCA plot of patients in the training set and test set.**


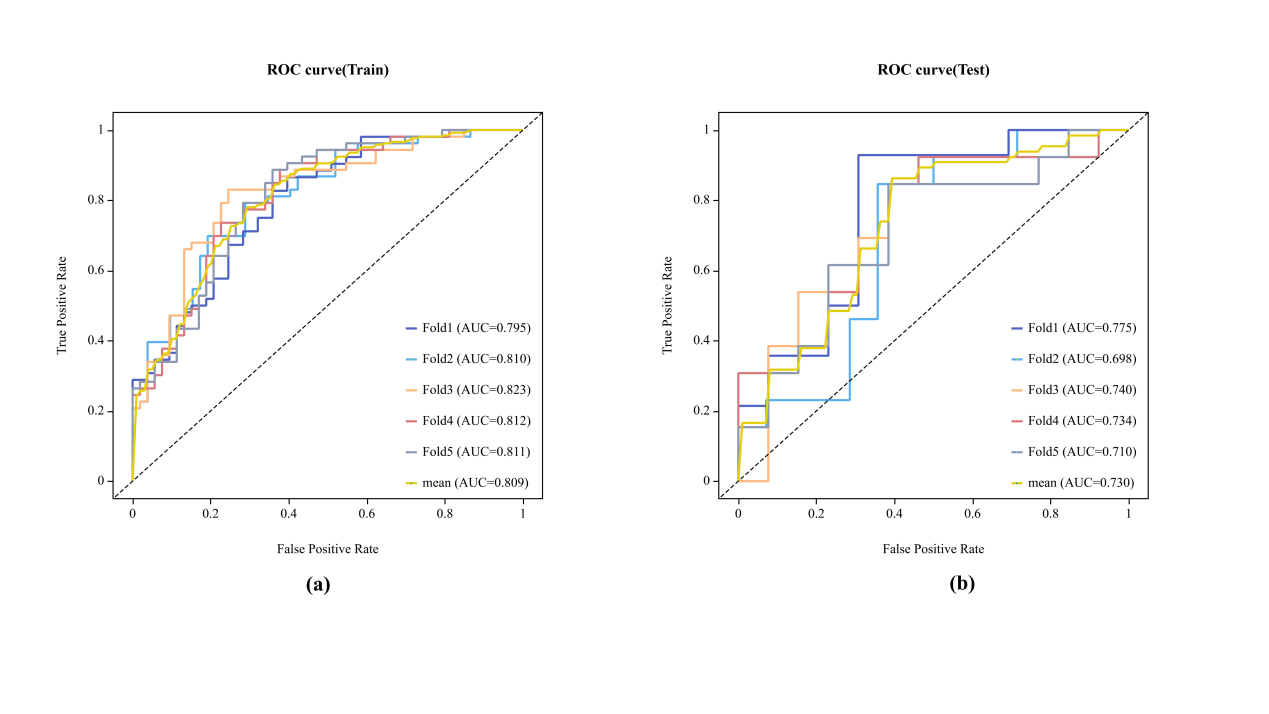


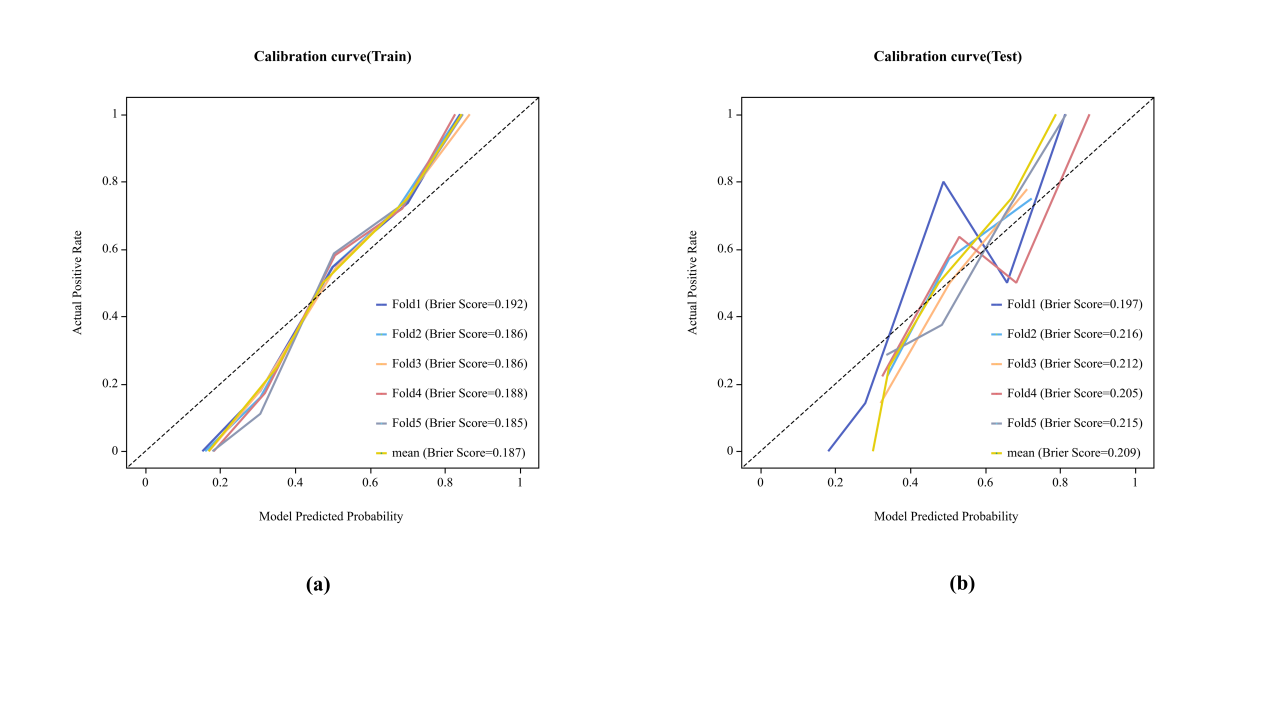


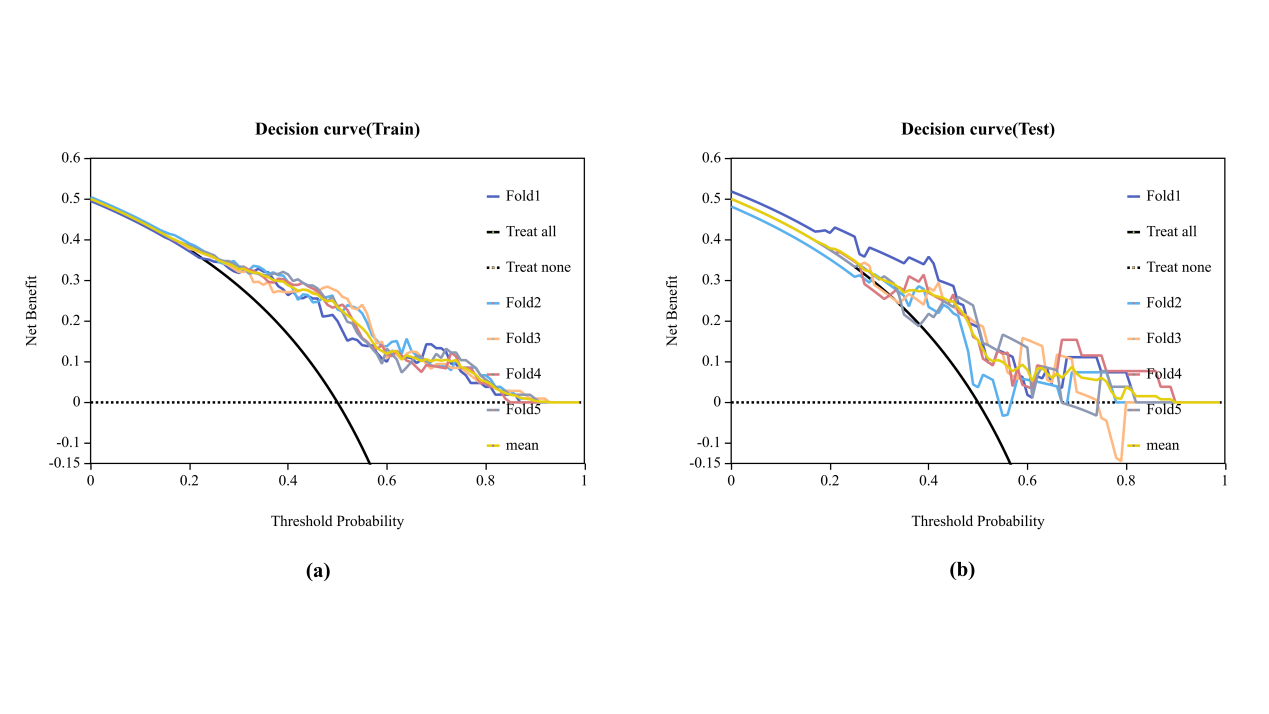


**Supplementary Table**

Supplementary Table 1**.** Baseline characteristics of the study population for the training set, test set, and external validation set

|  | Training set | | |  | | Test set | | |  | | External validation set | | |  | |
| --- | --- | --- | --- | --- | --- | --- | --- | --- | --- | --- | --- | --- | --- | --- | --- |
|  | SO  (n =53) | CTO  (n =52) | *P value* | | SO  (n =13) | | CTO  (n =14) | *P value* | | SO  (n = 50) | | CTO  (n = 50) | *P value* | |  |
| Clinical characteristics |  |  |  | |  | |  |  | |  | |  |  | |  |
| Male | 36 (67.9) | 37 (71.2) | 0.719 | | 8 (61.5) | | 13 (92.9) | 0.077 | | 41 (82.0) | | 42 (84.0) | 0.790 | |  |
| Age , y | 65 (55-70) | 63 (58-73) | 0.255 | | 62 (55-72） | | 63 (57-73) | 0.644 | | 63 (55-71) | | 59 (54-67) | 0.284 | |  |
| Body mass index, kg/m2 | 24.52 ± 3.51 | 24.21 ± 2.98 | 0.625 | | 23.78 ± 3.23 | | 23.12 ± 3.06 | 0.594 | | 24.17 ± 3.59 | | 23.84 ± 2.94 | 0.624 | |  |
| Hypertension | 31 (58.5) | 35 (67.3) | 0.350 | | 7 (53.8) | | 7 (50.0) | 1.000 | | 30 (60.0) | | 30 (60.0) | 1.000 | |  |
| Diabetes | 24 (45.3) | 19 (36.5) | 0.362 | | 2 (15.4) | | 3 (21.4) | 1.000 | | 20 (40.0) | | 16 (32.0) | 0.405 | |  |
| Smoking | 19 (35.8) | 24 (46.2) | 0.283 | | 1 (7.7) | | 4 (28.6) | 0.326 | | 14 (28.0) | | 18 (36.0) | 0.391 | |  |
| MI | 14 (26.4) | 17 (32.7) | 0.481 | | 0 (0) | | 3 (21.4) | 0.222 | | 4 (8.0) | | 5 (10.0) | 1.000 | |  |
| Unstable angina | 8 (15.1) | 13 (25.0) | 0.205 | | 2 (15.4) | | 3 (21.4) | 1.000 | | 13 (26.0) | | 11 (22.0) | 0.640 | |  |
| Stable angina | 12 (22.6) | 13 (25.0) | 0.777 | | 2 (15.4) | | 3 (21.4) | 1.000 | | 17 (34.0) | | 18 (36.0) | 0.834 | |  |
| Silent ischemia | 9 (17.0) | 8 (15.4) | 0.824 | | 0 (0) | | 2 (14.3) | 0.481 | | 16 (32.0) | | 13 (26.0) | 0.509 | |  |
| PCI |  |  |  | |  | |  |  | |  | |  |  | |  |
| PCI attempted | 45 (84.9) | 34 (65.4) | 0.021 | | 9 (69.2) | | 10 (71.4) | 1.000 | | - | | - |  | |  |
| Successful PCI | 43 (95.6) | 26 (76.5) | 0.029 | | 8 (88.9) | | 8 (10.0) | 1.000 | | - | | - |  | |  |
| Procedural time (min) | 5.00 (2.00-12.00) | 24.00 (15.25-41.000) | < 0.001 | | 2.50 (2.00-4.75) | | 18.50 (5.75-41.25) | 0.005 | | - | | - |  | |  |

Values are mean ± SD, median (25th and 75th percentile) or n (%).

MI = myocardial infarction; PCI = percutaneous coronary intervention.

Supplementary Table 2. CCTA features of the study population for the training set, test set, and external validation set

|  | Training set | |  | Test set | |  | External validation set | |  |
| --- | --- | --- | --- | --- | --- | --- | --- | --- | --- |
|  | SO  (n =53) | CTO  (n =52) | *P value* | SO  (n =13) | CTO  (n =14) | *P value* | SO  (n = 50) | CTO  (n = 50) | *P value* |
| Lesion location |  |  |  |  |  |  |  |  |  |
| LAD | 25 (47.2) | 17 (32.7) | 0.130 | 3 (23.1) | 2 (14.3) | 0.648 | 22 (44.0) | 22 (44.0) | 1.000 |
| LCX | 5 (9.4) | 7 (13.5) | 0.517 | 3 (23.1) | 4 (28.6) | 1.000 | 11 (22.0) | 6 (12.0) | 0.183 |
| RCA | 23 (43.4) | 28 (53.8) | 0.284 | 7 (53.8) | 8 (57.1) | 1.000 | 17 (34.0) | 22 (44.0) | 0.305 |
| Lesion length, mm | 8.90 (5.70-13.50) | 15.90 (11.10-24.30) | *<* 0.001 | 7.10 (3.60-9.30) | 11.95 (7.60-15.33) | 0.017 | 6.20 (3.48-9.13) | 11.75 (6.13-23.43) | *<* 0.001 |
| TAG (HU/10mm) | -1.26 (-2.43 to -0.21) | -0.04 (-2.15 to 0.11) | 0.101 | -1.67 (-2.96 to 0.06) | -0.82 (-3.42 to 0.09) | 0.583 | -1.79 (-2.97 to -0.82) | -1.14 (-2.21 to 0.14) | 0.054 |
| Blunt stump | 32 (60.4) | 42 (80.8) | 0.022 | 7 (53.8) | 11 (78.6) | 0.236 | 20 (40.0) | 36 (72.0) | 0.001 |
| Collateral vessel | 29 (54.7) | 21 (40.4) | 0.141 | 4 (30.8) | 6 (42.9) | 0.695 | 20 (40.0) | 26 (52.0） | 0.229 |
| Proximal branch | 25 (47.2) | 17 (32.7) | 0.130 | 6 (46.2) | 6 (42.9) | 1.000 | 14 (28.0) | 21 (42.0) | 0.142 |
| Distal branch | 11 (20.8) | 13 (25.0) | 0.604 | 8 (61.5) | 3 (21.4) | 0.054 | 8 (16.0) | 10 (20.0) | 0.603 |
| Bending>45° | 9 (17.0) | 12 (23.1) | 0.435 | 2 (15.4) | 2 (14.3) | 1.000 | 12 (24.0) | 16 (32.0) | 0.373 |
| Positive remodeling | 12 (22.6) | 19 (36.5) | 0.119 | 4 (30.8) | 3 (21.4) | 0.678 | 15 (30.0) | 24 (48.0) | 0.065 |
| Total plaque volume (mm^3^) | 307.99 (122.25 - 450.72) | 372.15 (230.79 - 571.94) | 0.089 | 141.46 (30.69 - 305.02) | 148.60 (6.08 - 492.81) | 0.961 | 233.48 (90.16 - 375.80) | 286.81 (81.82 - 460.10) | 0.605 |
| Calcified plaque volume (mm^3^) | 105.15 (17.30 - 205.65) | 93.61 (61.29 - 282.01) | 0.188 | 9.2 (6.14 - 167.69) | 54.38 (4.23 - 129.24) | 0.828 | 58.16 (12.8 - 152.11) | 59.60 (23.31 - 192.37) | 0.755 |
| Non-calcified plaque volume(mm^3^) | 197.88 (108.42 - 290.20) | 232.22 (123.10 - 340.85) | 0.159 | 71.45 (21.75 - 187.74) | 151.03 (31.96 - 379.15) | 0.624 | 154.91 (71.23 - 240.81) | 217.86 (78.44 - 342.69) | 0.516 |
| Low-attenuation plaque volume(mm^3^) | 35.71 (14.02 - 73.75) | 57.75 (21.56 - 97.42) | 0.118 | 7.49 (1.55 - 35.02) | 34.18 (10.70 - 66.18) | 0.384 | 36.25 (17.00 - 71.48) | 52.02 (18.73 - 103.56) | 0.090 |
| Calcified plaque load (%) | 35.53 (11.38 - 52.86) | 32.21 (21.03 - 51.59) | 0.756 | 28.85 (7.42 - 59.54) | 22.60 (9.43 - 40.72) | 0.663 | 32.62 (17.24 - 42.52) | 27.29 (8.32 - 53.58) | 0.516 |
| Non-calcified plaque load (%) | 197.88 (108.42 - 290.20) | 67.79 (48.41 - 78.97) | 0.745 | 71.15 (40.46 - 92.48) | 77.41 (59.28 - 89.61) | 0.253 | 67.38 (57.48 - 82.76) | 72.71 (46.43 - 91.68) | 0.214 |
| Low-attenuation plaque load (%) | 35.71 (14.02 - 73.75) | 57.75 (21.56 - 97.42) | 0.512 | 9.26 (1.82 - 17.37) | 13.82 (9.11 - 20.16) | 0.414 | 15.10 (10.26 - 25.33) | 19.95 (7.05 - 34.55) | 0.434 |

Values are median (25^th^ and 75^th^ percentile) or n (%).

**Abbreviations:** CTO=chronic total occlusion; SO=subtotal occlusion; CCTA=coronary computed tomography angiography.

Supplementary Table 3. The inter-observer agreement analysis for the CCTA image analysis

|  | κ Coefficient | 95%CI | *P value* |
| --- | --- | --- | --- |
| Lesion length, mm |  |  |  |
| Internal validation | 0..999 | 0.998,0.999 | <0.001 |
| external validation | 0.999 | 0.999,1.000 | <0.001 |
| TAG (HU/10mm) |  |  |  |
| Internal validation | 0.897 | 0.855,0.927 | <0.001 |
| external validation | 0.852 | 0.780,0.900 | <0.001 |
| Blunt stump |  |  |  |
| Internal validation | 0.982 | 0.975,0.987 | <0.001 |
| external validation | 0.956 | 0.934,0.970 |  |
| Collateral vessel |  |  |  |
| Internal validation | 0.849 | 0.784,0.894 | <0.001 |
| external validation | 0.847 | 0.773,0.897 | <0.001 |
| Proximal branch |  |  |  |
| Internal validation | 0.976 | 0.966,0.983 | <0.001 |
| external validation | 0.954 | 0.932,0.969 |  |
| Distal branch |  |  |  |
| Internal validation | 0.970 | 0.958,0.979 | <0.001 |
| external validation | 0.934 | 0.901,0.956 |  |
| Bending >45° |  |  |  |
| Internal validation | 0.961 | 0.945,0.973 | <0.001 |
| external validation | 0.960 | 0.941,0.973 |  |
| Positive remodeling |  |  |  |
| Internal validation | 0.958 | 0.941,0.971 | <0.001 |
| external validation | 0.914 | 0.870,0.943 |  |

Supplementary Table 4. Personalizing injection protocols with different concentrations of contrast and different weight.

|  | Weight（kg） | | | | |
| --- | --- | --- | --- | --- | --- |
| Contrast Concentration | <50 | 50～<60 | 60～<70 | 70～<80 | >80 |
| 320 mgI／ml | 38ml  3.8ml/s | 42ml  4.2ml/s | 50ml  5ml/s | 58ml  5.8ml/s |  |
| 350 mgI／ml | 35ml  3.5ml/s | 39ml  3.9ml/s | 46ml  4.6ml/s | 53ml  5.3ml/s | 56ml  5.6ml/s |
| 370 mgI／ml | 33ml  3.3ml/s | 37ml  3.7ml/s | 43ml  4.3ml/s | 50ml  5.0ml/s | 53ml  5.3ml/s |
| 400 mgI／ml | 30ml  3ml/s | 34ml  3.4ml/s | 40ml  4ml/s | 46ml  4.6ml/s | 49ml  4.9ml/s |

The IDR for different weight were 1.21gI/s, 1.36gI/s, 1.6gI/s, 1.85gI/s, 1.96gI/s respectively
